# Treatment of Disabled Sailors and Soldiers

**Published:** 1918-02-02

**Authors:** 


### The Hospital

The Workers' Newspaper of Administrative Medicine and Institutional Life, Administration, National Insurance and Health.

No. 1652, Vol. LXIII. SATURDAY, FEBRUARY 2, 1918. PRICE ONE PENNY

### TREATMENT OF DISABLED SAILORS AND SOLDIERS.

Whatever other responsibilities may emerge from the war it is certain that an undisputed place must be given to the men who, to their own loss, have stood in arms in defence of all our hearths and homes. To that great number who have died in this cause we can offer nothing but a national tribute of gratitude, and memories which will be preserved for generations. Their names, their deeds, their sacrifices—these will endure as examples of heroism and patriotism in the historical consciousness of a grateful people. We write them on a roll of honour so that in all time men may know those who readily gave even life itself for the sake of a great cause and in defence of a country they loved so well. It is with a salute to brave men that we recall their resting-place.

Not less worthy of honour and recognition are men who have indeed come back to us, but broken, maimed, crippled by wounds and suffering. These, also, have looked death in the face and suffered hardship on our behalf. They return with diminished powers to a life in which effort is a necessary condition of success. Upon each of them, in greater or less measure, weighs a handicap which they have suffered that others may be free. Let it not be forgotten that these men come from all ranks and conditions of our people and that they are of our own country and race and tongue. The pulse of youth still beats in their veins, and their natural outlook is to a future of achievement and happiness and purpose and success. In some degree this future is now limited, and in place of it appears doubt and restriction and anxiety. They must suffer strong and youthful competitors to whom blind fortune has granted a kindlier fate; and it may be that at times they fear the quality of endurance in the national gratitude. Fortunately on this point we believe we may reassure them. The national resolve is high that so far as possible these men shall be cared for, and that neither money nor skill shall be wanting to neutralise the sacrifices they have made for the common cause. Efforts to this end cannot be left to chance and to individual action. They must be organised and they must be national. The losses have been suffered for the country, and it is the country which must meet the obligation as one of honour and gratitude and good will.

The immediate responsibility created by this position is to be discharged by the Ministry of Pensions, and the country will demand that it be met with efficiency as well as with kindliness and good intentions. The mere payment of sums of money to men who, being no longer of military value, are discharged from the Army—important though such payments are—is only part of the task. For a longer or shorter period these men will need medical or surgical care. Moreover, by such agency a proportion of them may be restored to such a level of efficiency that they may, with advantage both to themselves and to the community, take a place among the workers of the nation. Here, obviously, comes an opportunity for the medical profession and for our voluntary hospitals; and we make no doubt that the opportunity will be welcomed. Co-operation of this order is indeed a necessity of the position. But let us again assert that the obligation and responsibility are truly national, and that neither an individual class nor individual institutions may be left to meet the position which belongs to the whole nation. Doctors and hospitals may stand in the forefront because of the special abilities they possess for the occasion. But they must act, not for themselves, nor for the classes and organisations they represent, but as the authorised and recognised ministers of a unanimous and grateful country. They may be the hands which carry help, but the motive power must be the resolve and co-operation of a whole people. Anything short of this would rob the undertaking of its real meaning and essential grace. The object is to meet gladly, and with a ready resolve, a claim which falls on all of us, and to be of full value every citizen must take his share in the discharge of the bond. The opportunity to do this is not equally great for all, but for each there is a place, and this place must be filled if the service of help and recognition to be offered to our disabled sailors and soldiers is to achieve its highest values as an expression of the nation's grateful and pious duty.

When the principles here presented come to be translated into practical action the argument may seem to take on a more mundane tone. But what we have to do is to recognise that the practical details of any scheme have an essential dignity when they are the expression of a high and appealing principle. To read (see p. 384) that the Ministry of Pensions and the British Hospitals Association have struck a bargain that so many shillings a day shall be paid for each man needing institutional treatment, may at first sight seem to have a cold commercial quality. But the agreement is the recognition—and there can be no other effective recognition—of the national character of the enterprise. By it the whole nation takes its position as the originator and supporter of the undertaking. These injured and disabled men are not to be made the recipients of charity. They are to be the honoured guests of the nation and all would fain serve them. To some of us is given the opportunity to serve them directly, but the welcome must be spoken by the voice of the nation. We welcome, therefore, the action of the British Medical Association, and of the Ministry of Pensions, which have issued to the secretaries of the honorary medical staffs of voluntary hospitals, and to the secretary of each Local War Pensions Committee respectively, full information of the terms of the arrangement come to. The arrangement of payments to the voluntary hospitals realises this demand in the most grateful form. The present scheme is tentative, as it should be, to enable it to become adequate as the result of experience of the requirements of the brave men and warriors disabled through war. The arrangement between the Ministry of Pensions and the voluntary hospitals, as the almoners of the nation, takes the right form to secure that it shall provide, to the fullest and most adequate extent, everything which the condition of each such patient, and his equipment for a useful and interesting life after his departure from the hospital, are found to require. The arrangement should demonstrate once more the adequacy and greatness of the principle on which the voluntary hospital system rests, and give further proof that in practice it represents the most efficient and desirable form of hospital administration which the world possesses.

